# Hydroethanolic Extracts of Raspberry (*Rubus idaeus*) Pomace as Ingredients of Functional Foods: Characterization and Effect of Gastrointestinal Digestion

**DOI:** 10.3390/plants14152444

**Published:** 2025-08-07

**Authors:** Ziva Vipotnik, Majda Golob, Alen Albreht

**Affiliations:** 1 Laboratory for Food Chemistry, Department of Analytical Chemistry, National Institute of Chemistry, Hajdrihova 19, SI-1000 Ljubljana, Slovenia; ziva.vipotnik@ki.si; 2 Institute of Microbiology and Parasitology, Veterinary Faculty, University of Ljubljana, Gerbičeva ulica 60, SI-1000 Ljubljana, Slovenia; majda.golob@vf.uni-lj.si

**Keywords:** antioxidative and antimicrobial activity, bioaccessibility, phenolic compounds, raspberry pomace

## Abstract

The extract of powdered raspberry pomace was characterized in terms of its phenolic profile and antioxidant and antimicrobial activity. Kuromanin, chlorogenic acid, protocatechuic acid, and pelargonidin-3-*O*-glucoside were found to be the major phenolic compounds, while the antioxidant activity of the extract correlated positively with the total phenolic content (TPC), which was 472.9 ± 0.1 mg GAE/g dw. The extract also showed good antimicrobial activity against Gram-positive foodborne bacteria. More importantly, in vitro bioaccessibility of phenols from the raspberry pomace extract was 5-fold higher when the extract was incorporated into meringue cookies. Although the concentrations of anthocyanins, flavonoids, and tannins decreased after the oral, gastric, and intestinal phases of digestion, the TPC slightly increased as the compounds were released from the food matrix. The content of available phenolics was 4-fold lower in the case of a commercial raspberry colorant, demonstrating that the waste from raspberry pomace could serve as a valuable health-promoting ingredient for functional food formulations.

## 1. Introduction

The valorization of food waste and food by-products is an important field of contemporary research, addressing both environmental and economic challenges. Scientific advancements in this field contribute to several Sustainable Development Goals (SDG3–good health and SDG12–responsible consumption and production) by facilitating a reduction in the carbon footprint and greenhouse gas emissions, mainly caused by the decay of different food wastes. These waste materials represent a natural and rich source of many nutrients, which can be recovered by using an appropriate combination of technological procedures such as drying, grinding, extraction, separation, and purification. Unfortunately, these materials are often still regarded as mere waste [1].

The raspberry (*Rubus idaeus*), with its typical appearance and flavor, has been known for centuries as one of the oldest fruits with medicinal properties. It is currently the fourth most important berry plant in the world. In the last 15 years, global raspberry production has increased from 373,000 t to 684,000 t [2,3]. In recent years, Serbia has become a leading producer, supplying the majority of raspberries sold worldwide and representing a rapidly growing segment of the local horticultural and food industry [4]. The majority of raspberries are processed into juices, jams, jellies, syrups, purees, wines, and other products [5]. However, the resulting pomace is normally discarded, which can lead to ecological and environmental problems due to its acidic nature and high moisture content. The raspberry pomace is an extremely rich source of bioactive compounds, namely proanthocyanins, anthocyanins, flavanols, and phenolic acids (especially ellagic acid). Evidence is accumulating that these compounds have health-beneficial properties, such as antioxidant and antimicrobial activity, as well as a broad range of physiological properties [6]. Anthocyanins are considered safe for human consumption, and they are categorized as a natural additive by the Codex Alimentarius Commission and carry the E-code E163 in the European Union. Therefore, food and beverage manufacturers can use anthocyanins as natural colorants to color foods, provided they meet the established specifications [7]. Nevertheless, there are no specific guidelines or standardized extraction protocols, as the strategy to procure the colorants will largely depend on the type of food source, on the targeted compounds, and on the end-application. However, in the face of the escalating climate change crisis, the research focus is now often directed towards sustainable procedures that are scalable and easily transferable to the industrial environment. Consistency and monitoring of feedstock are other challenges that producers of natural colorants are confronted with. Variability in total phenolic content (TPC) is observed even within specimens of the same plant species, influenced by both extraction techniques and cultivation environment, such as climate, proximity to the coast, and soil composition. Some plant species growing in colder climates, at higher altitudes, or in more arid environments often produce higher levels of these secondary metabolites than the same species growing under more favorable, mild conditions [4].

In this study, we evaluate the potential of a hydroethanolic raspberry pomace extract as a safe, easy-to-acquire, and health-promoting food additive for the preparation of pink-colored home-made functional foods. Its TPC, total flavonoid content (TFC), total anthocyanin content (TAC), and total condensed tannin content (TTC) were determined and correlated with the results of the in vitro antioxidant activity assay. The antimicrobial properties of raspberry pomace extract were tested against foodborne bacteria, while the bioaccessibility of phenolic compounds was estimated by using a static in vitro gastrointestinal digestion model. Finally, the obtained raspberry pomace extract was incorporated into the meringue cookie—a model functional food—to evaluate the impact of the heat-induced treatment on the extract’s bioactive compounds and demonstrate its practical advantages over commercial raspberry flavoring additives and colorants. This study not only contributes to sustainable waste management through the reuse of raspberry pomace but also provides new insights into the stability, bioactivity, and bioaccessibility of phenolics from confectionery products.

## 2. Results and Discussion

### 2.1. Phenolic Compounds (Flavonoids, Tannins, and Anthocyanins) from Raspberry Pomace Extract and Their Bioaccessibility

The chemical composition of foods and the antioxidant potential of their nutrients are among the decisive parameters when it comes to assessing the health-promoting effects of such products. The extraction processes, used to recover bioactive compounds from agricultural food waste, are controlled by different operational parameters, which can be tuned to favor specific groups of bioactive compounds [8]. Considering the influence of intrinsic and extrinsic factors as well as the different extraction methods used, the contents and profiles of phenolic compounds from raspberry can vary greatly. The study by Wu et al. [9] showed that the addition of up to 50% water to organic solvents increases the recovery of total phenols and flavonoids from raspberry pomace. In this work, several different water:ethanol (*v*/*v*) ratios were tested (50–100% ethanol _(aq)_), resulting in a TPC of 286.2 mg GAE/g dw with 50% ethanol _(aq)_, 472.9 mg GAE/g dw with 80% ethanol _(aq),_ and 253.7 mg GAE/g dw with 100% of ethanol, respectively. Therefore, 80% ethanol _(aq)_ was chosen as the optimal green, safe, and environmentally friendly extraction solvent that enables good extraction yields. Simplicity and accessibility are key features of this batch extraction method, easily transferable to any household or the industry, requiring no specialized extraction apparatus. However, certain challenges are to be expected when scaling up, such as the quality and reproducibility of the input pomace, drying and grinding consistency, and solvent recovery. Raspberry pomace extract contained high amounts of phenolic compounds (472.9 mg GAE/g dw), of which flavonoids were the most abundant among those tested, followed by anthocyanins and finally condensed tannins (Table 1).

A large portion of phenolic compounds is represented by phenolic acids (see Section 2.3), but other phenolic nutrients must also be present in the extract to account for the high TPC. In comparison, the methanolic extract of whole raspberries obtained by Vulić et al. contained 637.77 mg GAE/100 g TPC, 591.65 mg CE/100 g TFC, and 65.21 mg CE/100 g TAC [10]. These observed lower values, compared to the findings presented here, are likely a consequence of using fresh raspberry pomace that contains water, giving a lower apparent metabolite content. Moreover, our samples underwent grinding, thus increasing the available surface area for extraction and releasing the antioxidants from seeds, which are known to be rich in ellagitannins and flavan-3-ols. Moreover, the difference in reported values highlights the impact of the extraction solvent type on both extraction selectivity and overall yield. It is also important to recognize that the polyphenol composition within raspberry fruit itself is variable, influenced by factors such as location, fruit type, soil conditions, ripening stage, and weather patterns.

In another study investigating extraction solvents with varying water: ethanol ratios found that 50% aqueous ethanol yielded the highest phenolic contents: 29.52 mg GAE/g TPC, 32.99 mg RE/g TFC, and 0.27 mg CGE/g TAC [11]. In contrast, raising the ethanol concentration to 90% resulted in a 104% increase in TAC but a 17% reduction in TPC.

**Figure 1:**
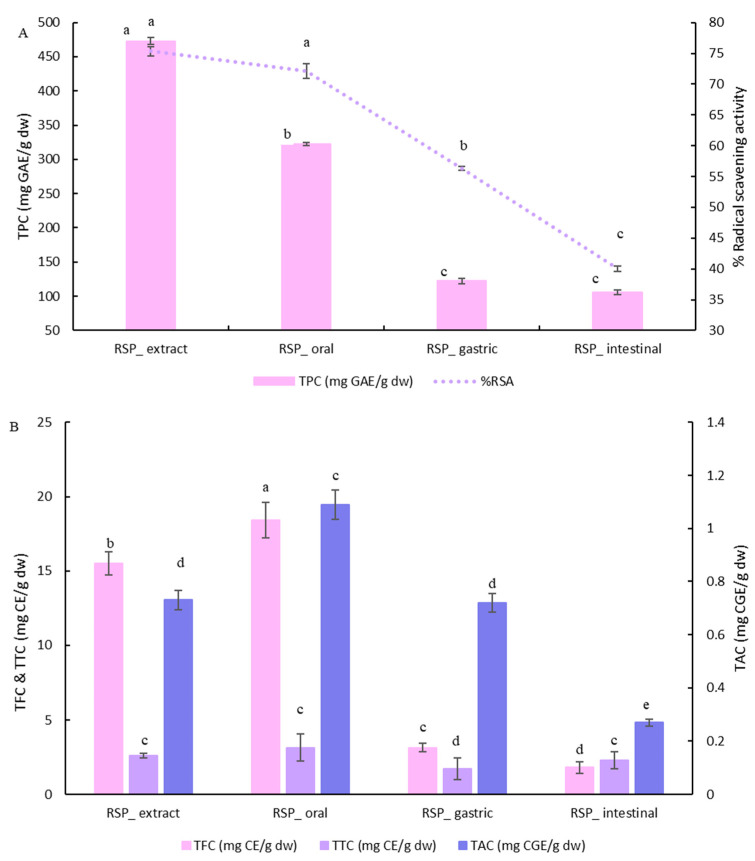
Total phenolic content and radical scavenging activity (% inhibition of DPPH) of raspberry pomace (RSP) extract (**A**), and TFC, TTC (left axis), and TAC (right axis) during digestion of RSP extract (**B**). Results are reported on a dry weight (dw) basis. Bars with different letters are significantly different (*p* ≤ 0.05).

During the in vitro digestion, TPC of the extract continuously decreased, resulting in 22.3% bioaccessibility of phenolics (Figure 1A). In contrast, TFC, TAC, and TTC increased after the first oral phase (Figure 1B), with the highest increase observed for tannins (33.3%), followed by anthocyanins (17.5%) and flavonoids (15.7%). Most of the flavonoid content was lost during the gastric phase (82.9%), which further decreased during the intestinal phase, resulting in a bioaccessibility of flavonoids of 11.7%. On the other hand, bioaccessibility of condensed tannins and anthocyanins was much higher at 88.4% and 37.1%, respectively.

According to previous reports [12,13], certain phenolic compounds may be sensitive to slightly alkaline conditions in the intestine, so they are likely to be structurally altered and/or degraded during the intestinal phase of digestion, resulting in low bioaccessibility. Each group of phenolic compounds can respond differently to mechanical pressures in the gut (e.g., peristalsis, churning) and to changes in the chemical environment as food moves from one gastrointestinal compartment to the next (pH, salt concentration, enzymes, etc.) [14]. For example, a non-related study reported that more than 65% of the total phenols and flavonoids in Chinese hawthorn were released during simulated oral digestion [15]. Podsędek et al. [16] investigated the effect of in vitro digestion on anthocyanins from red cabbage and the extract of red cabbage. Their findings were similar to those found here, showing that anthocyanin content and antioxidant capacity were lower in the final digest irrespective of the type of matrix [16]; however, a general increase in TPC was observed. A similar trend was reported by a recent study on raspberry pomace extract, supporting the findings presented here. TPC increased from 95.76 mg GAE/g to 108.39 mg GAE/g during digestion, while TFC and the antioxidant activity decreased by 13% and more than 50%, respectively [11]. Anthocyanins are generally considered to be most unstable at neutral or slightly basic pH as the colorless chalcone pseudobase is formed, which leads to the degradation of the anthocyanin chromophore [12,14,17]. Other phenolic compounds such as flavonoids and phenolic acids are usually more stable during digestion. In addition, the non-covalent intermolecular interactions between phenolic compounds and other components of the food matrix (such as proteins, digestive enzymes, bile salts, carbohydrates, lipids, dietary fiber, etc.) also alter their bioavailability (positively or negatively) [12]. Pinto et al. demonstrated that during the gastric phase, acidic pH and pepsin are the two main factors that contribute to the reduction in TPC by hydrolysis of certain polyphenols [18,19]. In addition, the poor solubility of some polyphenols at acidic pH may cause them to precipitate, rendering their recurrent dissolution very difficult, even in the alkaline environment of the intestine. As far as digestion in the intestine is concerned, the decrease in TPC can be explained by the change in pH and the action of pancreatin, as several polyphenols are unstable and degraded at neutral or alkaline pH. Under alkaline conditions, polyphenols can also bind to proteins via hydrophobic interactions, hydrogen bonds, or even form covalent bonds, leading to an apparent decrease in TPC [20]. In summary, predicting the dynamic changes in phenolic compound content from various foods during digestion is challenging due to the complex interplay of release and degradation, which are influenced by numerous variables. Our findings demonstrate that for raspberry pomace extract, both the total phenolic content and antioxidant activity decrease during digestion.

### 2.2. Antioxidant Potential of Raspberry Pomace Extract and the Effect of Digestion

The raspberry pomace extract showed promising antioxidant activity, irrespective of the antioxidant assay used (DPPH vs. FRAP); therefore, only the results from the DPPH radical scavenging are shown in the main text, while FRAP results are included in Supplementary Material (Table S1). The radical scavenging activity (RSA) decreased steadily from 75.3% in the initial extract to 40% following the final intestinal digestion stage (Figure 1A). This decrease in antioxidant activity correlates with the TPC but not perfectly, indicating a complex scenario where the release and degradation of phenolics, alongside their differing redox potentials (and thus antioxidant effectiveness), contribute to the overall change. Plant seeds often contain a relatively large amount of insoluble phenols, which may contribute to the overall bioactivity of the food if the seeds are ruptured and the contents are released during digestion [21].

### 2.3. Bioaccessibility of Selected Polyphenol Constituents of Raspberry Pomace Extract

The number and diversity of natural phenolic compounds in raspberry pomace are high. This diversity originates not only from the different compound classes, but also from the numerous ways in which they are naturally modified by glycosylation and acylation at different positions within the molecules. Here, the most abundant phenolic compounds from the raspberry pomace extract were identified and quantified by targeted HPLC-DAD (Table 2).

Kuromanin (also known as cyanidin-3-*O*-glucoside, asterin, or chrysanthemin) and chlorogenic acid were the most abundant compounds in the extract with concentrations of 72.9 mg/g and 43.9 mg/g, respectively, followed by protocatechuic acid (29.1 mg/g) and pelargonidin-3-*O*-glucoside (21.8 mg/g). The remaining phenolic compounds were mainly phenolic acids (coumaric acid, ellagic acid, ferulic acid, syringic acid, gallic acid) and two polyphenols (catechin and myricetin), for which the concentrations ranged from 11 to 17 mg/g.

During digestion, the highest losses were observed for the anthocyanins kuromanin and pelargonidin-3-*O*-glucoside. Caffeic acid was detected only after the intestinal phase (2.3 mg/g), most likely due to the chemical or enzymatic hydrolysis of chlorogenic acid (Figure S1) [22]. Similarly, the increase in gallic acid from 12.7 mg/g to 35.3 mg/g could at least in part be explained by the in situ transformation of hydrolysable gallotannins during digestion [23]. Gallic acid has also been reported as a breakdown product of anthocyanins, in particular cyanidin-3-*O*-glucoside [24]. This increased release of phenols can be attributed to the emulsifying properties of bile salts, which facilitate the breakdown of the non-covalent complexes that form between these bioactive molecules and gastric enzymes, particularly pepsin, thereby improving their solubility and accessibility. In addition, flavonoid molecules are readily transformed to other small metabolites in the intestine (e.g., phenolic acids), thus modulating the number and activity of antioxidant molecules [25].

Although ellagic acid content in the non-treated sample (16.3 mg/g) was lower than that of the most abundant compounds, this observation is consistent with the existing literature [4] where the highest amount of ellagic acid (about 50% of total phenols) was found in the whole raspberry fruit, and 35% of those were found in seeds. As already mentioned, an important factor that influences the bioavailability of polyphenols is the nature of the plant matrix. The plant cell walls represent a barrier to digestion [26]. When plant cells are ruptured (by chewing or crushing), the released phenolic compounds do not necessarily increase the antioxidant activity and TPC, as they also bind to dietary fiber, leading to a modulation of their relative bioavailability. Polyphenols encapsulated or bound to dietary fibers are both poorly extractable and poorly soluble in gastrointestinal fluids [27]. Nonetheless, results in Table 2 show that most phenolic compounds from the extract of raspberry pomace have good bioaccessibility.

### 2.4. Antimicrobial Activity of the Raspberry Pomace Extract

Foodborne bacteria pose a significant threat to food safety. The World Health Organization estimates that annually, 600 million people fall ill due to food poisoning. These pathogens can impact the quality of food at all stages of the production chain and storage. In the present study, the antimicrobial activity of raspberry pomace extract was preliminarily evaluated against eight common foodborne bacterial strains by the disk diffusion method (Table 3).

The extract demonstrated clear inhibitory zones in all Gram-positive and Gram-negative bacteria with the exception of *S.* Enteritidis, which showed no inhibition. The findings align with previous studies, which indicate that raspberry extracts exhibit stronger antimicrobial activity against selected Gram-positive bacteria and Gram-negative bacteria [28]. Similar to the present study, inhibitory effects on *S. aureus* and *L. monocytogenes* have been found. However, differences between studies, particularly when considering *S.* Enteritidis, *E. coli*, and *Enterobacter aerogenes*, could be attributed to variations in extraction methods, solvent types, and the geographical origin of raspberry samples. These methodological differences underscore the importance of standardizing protocols in future studies to allow for relevant comparison.

### 2.5. Bioactive Properties and Bioaccessibility of Phenolic Compounds from Meringue Cookies Fortified with Raspberry Pomace Extract or with a Commercial Raspberry Colorant

**Figure 2:**
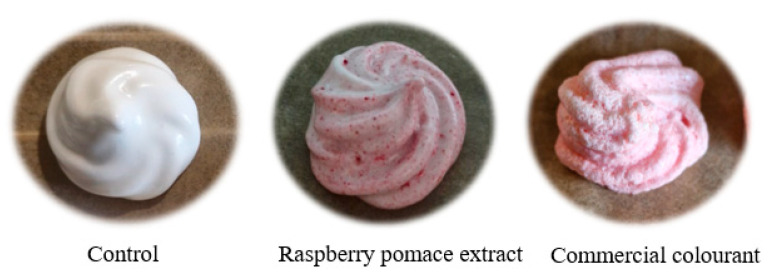
Sample of meringue cookie prepared in study (**left**—control cookie, **middle**—cookie with raspberry pomace extract, **right**—cookie with commercial colorant).

Natural alternatives to artificial food additives are being sought to circumvent the potential harmful effects of synthetic products and promote innovation in the food industry. However, synthetic additives are still often preferred due to certain advantages. For example, synthetic food colorants are generally more stable and retain their properties more efficiently during their preparation and incorporation into foods, heat treatment, and finally storage. Therefore, when choosing a naturally derived colorant, the characteristics of the source and its processing prior to consumption should be carefully considered in order to achieve the desired food-coloring effect and taste. Several studies have already reported on successful replacements of synthetic dyes with natural ones in the case of yogurts [29], candies [30], and tagliatelle pasta [31], among others. Here, we focus on a natural, healthy, and eco-friendly alternative to an artificial raspberry food additive that is used as a colorant as well as a fragrance and taste enhancer of foods. To demonstrate its applicability, the extract of raspberry pomace was added to the dough from which meringue cookies were prepared as a model food. Meringue is a well-known confectionery product made from simple ingredients, namely beaten egg whites and sugar, sometimes with the addition of acidic ingredients. The foamy properties of the egg whites are the basis for the characteristic texture of the meringue. Whipping breaks the hydrogen bonds of the albumin protein in the egg whites and produces a white, thin-felt foam with air pockets, while added sugar and acidic compounds stabilize and strengthen the foam. Sugar also acts as a stabilizer [32]. This type of food was purposely selected because its preparation involves a heat treatment at 70 °C, which could trigger changes in the color and bioactivity of the end-product (meringue cookie). Three different batches of meringue cookies (Figure 2) were prepared: (a) cookie control, (b) cookies containing raspberry pomace extract, and (c) cookies containing commercial raspberry colorant. The antioxidant properties of the cookies were evaluated (Table 4), and TTC, TFC, TPC, and TAC were determined.

The results for control cookies are also shown in Table 4, with the background subtracted from both fortified cookies for easier comparison. The TPC of cookies prepared with raspberry pomace extract remained relatively stable throughout the digestion period. Nonetheless, a slight increase from 7.6 mg/g to 10.8 mg/g was observed after the last two digestion phases. The increased phenol content in the gastric mash is probably due to the digestion of cookies under acidic conditions (pH 3), the presence of gastric enzymes, and a longer contact time, which allows the phenols to be released from the matrix. The increase observed after the intestinal phase most likely reflects the action of the pancreatin-bile salts at pH 7, which facilitates the release of polyphenols bound to the matrix [18]. The concentration of flavonoids in the undigested sample was quantified at 1.2 mg CE/g dw and demonstrated a 57% reduction during the oral phase, but a 36% increase during the intestinal phase, resulting in 59.5% bioaccessibility. Anthocyanins were detected only in very small amounts (expressed as mg/100 g), but their content increased after the gastric phase, likely due to the breakdown of the food matrix at the lower gastric pH. There is a possibility that anthocyanins could bind to components of the pancreatin and/or bile salt mixture in subsequent digestive phases, potentially affecting their later detection. Co-digestion with other common foods could potentially help protect the labile anthocyanins without markedly reducing the level of other bioavailable polyphenols from food [33], but this was out of the scope of the current study.

The content of condensed tannins in undigested cookies was determined at 0.71 mg/g and it decreased consistently with the digestion, affording a bioaccessibility of 28%. In contrast, no tannins were detected in meringue cookies made with the commercial raspberry colorant. The concentration level of TPC (0.7 mg/g) was about 10-fold lower in comparison to the extract-fortified cookies, but increased during digestion to 2.9 mg/g. This increase is not particularly surprising since the commercial raspberry colorant also contained whole condensed raspberries, which could release certain nutrients only upon digestion. TFC changed only slightly during digestion from 0.21 mg/g to 0.17 mg/g. Although cookies with commercial raspberry colorant showed a higher bioaccessibility of phenolic compounds, cookies fortified with the raspberry pomace extract contained nearly 4-fold higher absolute TPC after digestion, representing the actual content of bioactive phytonutrients that are available for absorption by the epithelial cells.

A control experiment exposed both the crude raspberry pomace extract and extract-infused meringue cookie dough to 70 °C for 2 h to assess the protective effect of the meringue matrix (Table S2). While total phenolic recovery was comparable, flavonoids and anthocyanins showed significantly higher recovery in the cookie. Notably, anthocyanins were well-preserved by the cookie matrix (93% recovery), unlike their complete degradation in the crude extract. Hydrolysable tannins were stable in the crude extract (92% recovery), but only 50% were recovered in the cookie. The reduction in tannins likely stems not from their degradation, but from their strong interaction with cookie (egg) proteins, forming complexes that are known to precipitate or aggregate.

The amount and number of phenolic compounds released from food may vary with the composition of the food, its processing method, the content of water, and the interaction of the phytochemicals with any other co-administered foods. Understanding food digestion, particularly its disintegration to release nutrients for homeostasis, is crucial. This improved understanding reveals that the food matrix structure significantly influences the kinetics of macronutrient transit and hydrolysis, thereby impacting overall nutrient bioavailability [34]. Several studies indicate that the extractability of polyphenols improves in the presence of fat, milk, egg proteins, and gelatins [25,31,32]. The inclusion of mugwort extract in various food ingredients (coconut oil, egg white albumen, brown rice powder, inulin, and mixtures thereof) revealed that oil significantly enhances polyphenol bioaccessibility (62.9%) and antioxidant activity after in vitro digestion. Conversely, egg white albumen had the strongest negative impact, reducing polyphenol bioaccessibility to just 12.49% [35]. Similarly, the availability of polyphenols from blackberries after an in vitro digestion is reduced by 68% [36]. On the other hand, TPC and TFC after gastrointestinal digestion of broccoli sprout extracts showed 122% and as much as 190% recovery, respectively, underlining the importance of the functional food matrix [37]. While enhanced polyphenol bioavailability offers health benefits, polyphenol-rich foods can also present negative implications. It is known that the binding of polyphenols to proteins reduces the digestibility of proteins, either by interacting with digestive enzymes or by protecting dietary proteins from degradation by enzymes [38]. In accordance with the results presented here, Tomas et al. showed that despite significant losses of total phenolics (88%), flavonoids (89%), anthocyanins (97%), and antioxidant capacity (88–93%) during jam processing of black mulberries, the bioaccessibility of total phenolics, and anthocyanins from this sugar-rich product significantly increases by 16%, 12%, and 37%, respectively [39]. The heat treatment of foods might improve the bioaccessibility of polyphenols due to the disruption of plant tissues and polyphenol–polysaccharide complexes. However, the effect can be double-edged as elevated temperatures can also lead to thermal degradation of phenolic compounds [40]. Moreover, it was shown that, depending on the reducing sugar type, Maillard products can form when meringue cookies are baked at elevated temperatures [41], which can interact with polyphenolic compounds. However, since meringue cookies must remain white, they are baked below the Maillard reaction’s typical onset temperature of 140 °C to prevent browning.

The antioxidant activities of meringue cookies correlate quite well with the phenolic content and show that the food matrix efficiently encapsulates and protects the content as well as the bioactivity of the raspberry pomace extract. The undigested sample showed 30.3% scavenging capacity, which was generally preserved during the digestion, increasing slightly towards the end to 37.7%. This increase could be attributed to the antioxidants being released from the food matrix during processing. It should be stressed that the antioxidant behavior of the extract incorporated within cookies was the opposite of that of the extract alone, where the antioxidant activity decreased steadily throughout digestion. Cookies with the commercial raspberry colorant showed consistently about 4-fold lower antioxidant activity. Moreover, Awatsuhara et al. [42] reported that the combination of egg white proteins with natural polyphenols can increase antioxidant activities during thermal processing. Antioxidants do not contribute to the flavor profile of foods; however, they are known to protect against various chronic diseases caused by oxidative stress [43]. Another important aspect of food digestion is the breakdown of food during digestion, which leads to the release of nutrients that are available to maintain homeostasis. A better understanding of the mechanisms underlying digestion is improving our knowledge of the kinetics of macronutrient hydrolysis and bioavailability, and thus the bioavailability of nutrients; therefore, the structure of the food matrix plays a key role in the kinetics of macronutrient transit and hydrolysis.

## 3. Materials and Methods

### 3.1. Extraction of Phenolic Compounds from Raspberry Pomace

The optimally ripe raspberry fruits (*Rubus idaeus ‘Rossana’*) were harvested near Žalec (46.253498, 15.151446), Slovenia, during August 2023. They were initially squeezed into juice using the pressing technique, leaving behind the raspberry pomace. The pomace was lyophilized for 36 h using a VirTis Benchtop Pro 9L ES freeze dryer from SP Industries Inc. (Warminster, PA, USA). The lyophilized pomace was then ground into powder with a coffee blender (Gorenje, Velenje, Slovenia). The hydroethanolic extract was prepared by maceration of the powdered pomace (1 g) in 30 mL of ethanol-water (8:2, *v*/*v*) at 25 °C (magnetic stirring at 150 rpm for 1 h) and subsequently filtered through Whatman No. 4 paper. The solid residue was then extracted once more by using 30 mL of the same solvent mixture. The combined extracts were filtered through 0.45 µm of polyvinylidene fluoride (PVDF) filter, and ethanol was removed under reduced pressure at 40 °C. The remaining aqueous solution was lyophilized to obtain 702.41 mg of dry mass. The obtained extract was stored in a glass amber vial at −20 °C in the dark until further use.

### 3.2. Determination of Chemical Constituents in Raspberry Pomace Extracts

Unless stated otherwise, assays were carried out by using 1 mg of dry raspberry pomace extract (RSP) (Section 3.1), which was dissolved in 1 mL 80% ethanol _(aq)_. When necessary, this solution was further diluted with the same solvent.

#### 3.2.1. Total Phenolic Content

The TPC of the pomace extracts was evaluated by the colorimetric Folin–Ciocalteu assay [44]. Briefly, 5 μL of sample (Section 2.2) was mixed with 60 μL of Na_2_CO_3_ (7.5%), 15 μL of Folin–Ciocalteu reagent (Supelco, Sigma-Aldrich, St. Louis, MO, USA), and 200 μL of water, and the mixture was kept at 40 °C for 15 min. The absorbance was measured at 760 nm using a spectrophotometric microplate reader (Synergy HT, BioTek Instruments, Inc., Winooski, VT, USA). Solutions of gallic acid (2–100 mg/L, R^2^ = 0.99) were used for calibration and TPC was expressed as milligrams of gallic acid equivalent per gram of dry weight of raspberry pomace extract (mg GAE/g dw).

#### 3.2.2. Total Flavonoid Content

The TFC of the raspberry pomace extract was determined by the aluminum chloride colorimetric method according to Chandra et al. [45]. Briefly, 0.25 mL of the reconstituted extract (Section 2.2) was mixed with 1 mL of water and 0.75 mL of NaNO_2_ (5%). After 6 min, 0.75 mL AlCl_3_ (10%) was added, and 1 mL of NaOH (4%) was added after an additional 6 min, and filled up to 2.5 mL with water and vigorously shaken. Solutions of catechin (2–100 mg/L, R^2^ = 0.99) were used for external calibration purposes, and TFC was expressed as milligrams of catechin equivalent per gram of dry weight of raspberry pomace extract (mg CE/g dw).

#### 3.2.3. Total Condensed Tannin Content

The TTC was determined by the method of Broadhurst et al. [46] with a slight modification described by Rebaya et al. [47], using catechin as an external calibration standard in the range 2–100 mg/L (R^2^ = 0.99). To 400 µL of reconstituted extract (Section 2.2), 3 mL of vanillin solution (4% in methanol) and 1.5 mL of concentrated hydrochloric acid were added. After 15 min of incubation at RT the absorbance was read at 500 nm. The TTC was expressed as milligrams of catechin equivalent per gram of dry weight of raspberry pomace extract (mg CE/g dw).

#### 3.2.4. Total Monomeric Anthocyanin Content

The TAC of the raspberry pomace extract was determined by using the pH differential method, as described in the Association of Official Agricultural Chemists (AOAC) Method 2005:07 [48]. The absorbance was measured spectrophotometrically against a blank sample at 520 nm as well as 700 nm, prepared in buffer solutions at pH 1.0 and pH 4.5. The TAC was calculated according to Equation (1), and the results are reported as milligrams of cyanidin 3-glucoside equivalents per 100 g of dry weight of raspberry pomace extract (mg CGE/100 g dw).

TAC = (A × MW × DF × 10^3^)*/*(ε × l) (1)

where A = (A_520 nm_ − A_700 nm_) pH1.0 − (A_520 nm_ − A_700 mn_) pH4.5; MW (molecular weight) = 449.2 g/mol for CGE; DF = dilution factor; 10^3^ = unit conversion (g to mg); ε = molar extinction coefficient (26,900 l × mol^−1^ × cm^−1^); and l = optical pathlength in cm.

#### 3.2.5. Chromatographic Analysis of Phenolic Compounds

The method described by Ferreira-Santos et al. was used for qualitative and quantitative analysis [44]. Briefly, the reconstituted extracts (Section 3.2) were filtered through a 0.22 μm PVDF filter. The analysis was performed by a high-performance liquid chromatographic system equipped with a diode array detector (HPLC-DAD; Dionex Ultimate 3000, Thermo Scientific, San Jose, CA, USA). Separation was performed on a reversed-phase Acquity UPLC BEH C18 column (2.1 mm × 100 mm, 1.7 μm) from Waters (Milford, MA, USA) at 35 °C. The mobile phase consisted of solvent A (0.1% formic acid) and solvent B (0.1% formic acid in acetonitrile). The following gradient was applied: 5% to 70% B in the first 17 min, followed by a linear gradient from 70% to 5% B in 3 min, then 5% B was held for 10 min. The injection volume was 2 μL, and the flow rate was set to 0.27 mL min^−1^. The annotation of compounds was performed by comparison of retention times and UV–vis spectra with reference standard materials (Extrasynthèse, Genay, France). Different acquisition wavelengths were used: 280 nm for hydroxybenzoic acids (gallic, protocatechuic, and syringic acid) and ellagic acid, 320 nm for hydroxycinnamic acids (caffeic, chlorogenic, coumaric, ferulic, and synaptic acid), 360 nm for flavonoids (myricetin, catechin), and 520 nm for anthocyanins. Quantification was achieved by the external standard calibration method, using a corresponding phenolic standard. The data were acquired and processed using Xcalibur software version 4.2.47 (Thermo Finnigan, San Jose, CA, USA).

### 3.3. Bioactivity of Raspberry Pomace Extracts

#### 3.3.1. Antioxidant Assays

Antioxidant activity was measured by the 2,2-diphenyl-1-picrylhydrazyl (DPPH) (Sigma-Aldrich, Burlington, MA, USA) and FRAP antioxidant assay (Method S1). Reconstituted extract (Section 3.2) was diluted in series with 80% ethanol _(aq)_ to obtain solutions at eight different concentration levels.

The DPPH colorimetric reaction was carried out in 96-well microplates containing 20 µL of the diluted extracts and 200 µL of the reagent solution. DPPH reagent solution consisted of 7.9 mg DPPH dissolved in 80% methanol _(aq)_ to obtain an absorbance value of 0.70 ± 0.02 at 517 nm. The mixed reagent and sample solutions were allowed to stand for 30 min at room temperature (RT) in the dark. Afterwards, the absorbance was measured at 517 nm. A calibration curve was prepared using standard solutions of gallic acid (1–200 mg/L, R^2^ = 0.99). The radical scavenging activity was calculated by the DPPH percent inhibition (Equation (2)) and expressed as micromoles of gallic acid equivalents per g of dry weight of raspberry extract (μmol GAE/g dw).

% Inhibition of DPPH = (1 − AC/AS) × 100(2)

where AS is the sample absorbance and AC the absorbance of the control sample.

#### 3.3.2. Antimicrobial Activity of Raspberry Pomace Extract

The antimicrobial activity of the extract was assessed with the agar disk diffusion test method using different Gram-negative and Gram-positive strains from an internal bacterial collection at the Institute of Microbiology and Parasitology, Veterinary Faculty Ljubljana. The potential antimicrobial activity of raspberry pomace extract was investigated against the following foodborne bacterial strains: *Bacillus cereus* (RDK 056, ATCC 25923), *Escherichia coli* (RDK 052, ATCC 25922), *Klebsiella pneumoniae* (RDK 070A, ATCC 51503), *Listeria monocytogenes* (RDK 059, Wurzburg), *Pseudomonas aeruginosa* (RDK 184, ATCC 27853), *Salmonella* enterica subsp. *enterica serovar* Enteritidis (RDK 055, CAMP 5439), *Staphylococcus aureus* (RDK 056, ATCC 25923), and *Yersinia enterocolitica* (RDK 058, ATCC 9610).

A bacterial suspension equivalent in density to the 0.5 McFarland turbidity standard was prepared and spread on Mueller–Hinton agar plates (Sigma-Aldrich). After that, the Whatman AA disc (diameter 6 mm) containing 10 µL of extract (1 mg/mL in ethanol-water 8:2; *v*/*v*) was placed on the inoculated agar and incubated at 37 °C for 24 h. The tetracycline disk (30 µg, BBL Becton Dickinson, Franklin Lakes, NJ, USA) was used as a positive control, and the disk containing 10 µL of solvent (ethanol-water 8:2; *v*/*v*) was used as a negative control. After incubation, the inhibition zones (the growth-free zones around the disk) were measured in mm using a vernier caliper. Each bacterial strain was tested in duplicate, and the data are given as the mean values with the standard deviation.

### 3.4. Preparation of Meringue Cookies

A total of 65 g of pasteurized liquid egg white, acquired from DESPAR (Amsterdam, The Netherlands) albume d’uovo (composition per 100 g: 182 kJ or 43 kcal; 0.9 g of carbohydrates, without sugars; 9.8 g of protein; and 0.42 g of salt), was mixed with 0.20 g of salt and 100 g of powdered granulated sugar (composition per 100 g: 1700 kJ; 100 g of carbohydrates—all sugars) with a stand mixer (TEFAL Masterchef Gourmet, Rumilly, Haute-Savoie, France), at maximum speed, for 10 min. The recorded relative ambient humidity was 30%. The additives were added subsequently and mixed by hand. Three different dough batches were separately prepared: (a) control meringue cookie dough with no additive present; (b) meringue cookie dough with commercial raspberry aroma/colorant “Aroma ETOL” (composition: water, ethanol, raspberry aroma, food color E122 (azorubine); 2 mL per 100 g meringue dough); and (c) meringue cookie dough with raspberry pomace extract (2 mL (0.03 g/mL) per 100 g meringue dough). The commercial coloring additive was dosed according to the manufacturer’s recommendations, while the raspberry extract (prepared in this study) was added in an amount designed to replicate the color of the meringue cookie fortified with the commercial colorant.

For each batch of the meringue cookies, the dough was put into a pastry bag fitted with a piping tip and spread on a tray lined with parchment paper and dried for 80 min at ≈70 °C. Afterwards, the meringue cookies were left to cool inside the oven for approximately 2 h before being stored at room temperature and in the dark until further analysis.

### 3.5. In Vitro Digestion of Raspberry Pomace Extract and Meringue Cookies

Lyophilized extracts of raspberry pomace and meringue cookies were subjected to INFOGEST 2.0 harmonized in vitro digestion, which simulates the human digestion process. The protocol was previously described by Brodkorb et al., which includes the determination of activities of relevant enzymes [49]. In order to obtain measurable concentrations of the bioactive compounds at the end of all the phases of digestion, the dried extracts were dissolved in ultrapure water to a concentration of 30 mg/mL. In the case of cookies, 5 g of slightly crushed (approximately 1 cm particle size) and homogenized material was used. The digestion process started with the oral phase by adding 4 mL of simulated salivary fluid (SSF) at pH 7, α-amylase (activity of 75 U/mL) (Sigma-Aldrich; A1031), and filling to 10 mL with water. The solution was incubated at 37 °C and rotated at 45 rpm using a Benchtop Tube Rotator (Cole-Parmer Instrument Company, Vernon Hills, IL, USA) for 5 min. For the next gastric phase, 8 mL of simulated gastric fluid (SGF) and pepsin (activity of 2000 U/mL) (Sigma-Aldrich; P7012) were added to the oral mixture. The mixture was acidified to pH 3 using 4 M HCl and then filled with water to 20 mL. The mixture was incubated for 120 min (37 °C and 50 rpm), keeping the pH constant and adjusting it if needed every 30 min. After 120 min, 20 mL of simulated intestinal fluid (SIF) was added, containing pancreatin (activity of 100 U/mL) (Sigma-Aldrich; P7545) and bile salts (80 mg/mL) (Sigma-Aldrich; B8381). The pH of the final intestinal mixture (cca 40 mL) was adjusted to 7 using NaOH (1 M) and incubated for 120 min (37 °C and 50 rpm). Upon completion of the digestion, the samples obtained were centrifuged (10 min at 2500 g) and filtered (PVDF 0.45 µm) for further analysis. In the case of the meringue cookies, the Amicon Ultra-15 Centrifugal Filter (50 kDa, Sigma-Aldrich) was used for the filtration of samples. Each digestion phase was performed in triplicate, and controls/blanks were obtained by adding water instead of extracts. The bioaccessibility of compounds was determined by using Equation (3):

Bioaccessibility (%) = (A/B) × 100(3)

where A is the compound content in the digesta and B is the compound content in the non-treated sample.

### 3.6. Statistical Analysis

All statistical analyses and data visualizations were performed with the statistical software R 4.2.1 (Boston, MA, USA). Experiments were performed in triplicate, and results were expressed as mean ± standard deviation (SD). Differences were analyzed by analysis of variance (ANOVA) followed by Tukey’s HSD test, and the statistical significance level was set at *p* < 0.05.

## 4. Conclusions

The issue of waste in global fruit and vegetable production, nearing 50%, underscores the importance of valorizing agro-industrial by-products. This research demonstrates a viable solution by showing that raspberry pomace from juice production can be effectively reutilized. Hydroethanolic extraction of this pomace yielded a phenol-rich extract with notable antioxidant and antimicrobial properties. The simulated in vitro digestion process facilitated the release of phenolic compounds, primarily in the oral and gastric phases. To understand the transformations of the main antioxidants during digestion, we monitored the content of fourteen most abundant phenolic compounds, enabling the proposition of key conversion pathways. The final digesta still showed good antioxidant activity relative to the untreated extract, showing a slight decline that is consistent with both the release patterns and the chemical transformations undergone by antioxidants during digestion. While replacing commercial raspberry colorant with raspberry pomace extract did not yield a more intense pink hue or a stronger raspberry aroma in meringue cookies, this substitution led to notable improvements in nutritional content and antioxidant activity. Specifically, the cookies enriched with the natural pomace extract contained approximately 4 to 12 times more phenolics than those using the artificial additive, and this difference was maintained throughout the simulated digestion process. Bioaccessibility of antioxidants from cookies was higher in comparison with the crude extracts, most likely due to the protective effect of the cookie matrix on the bioactive compounds. The results highlight raspberry pomace extracts as good sources of bioactive compounds, positioning them as valuable food additives. The inclusion of pomace extract not only gives the intended pink color but also improves the antioxidant capacity and nutritional profile of food products, thus providing noteworthy health advantages. Nonetheless, before such a solution could be introduced into the market, several additional tests should be carried out, such as the sensory analysis of the final product and in vivo confirmation of bioactivity of the health-promoting compounds.

## Figures and Tables

**Table 1:** The total amount of phenolic compounds of raspberry pomace extract by compound group. Results are expressed as mean ± SD of triplicate determinations.

Sample	TPC (mg GAE/g dw)	TFC (mg CE/g dw)	TAC (mg CGE/g dw)	TTC (mg CE/g dw)
Raspberry pomace extract	472.9 ± 0.1	15.5 ± 0.1	2.6 ± 0.2	0.73 ± 0.01

**Table 2:** Content of most abundant phenolic compounds from raspberry pomace extract. Quantification was performed by the external standard calibration method, and the values are given as mean ± SD of triplicate determinations.

Compound	Concentration (mg/g)		Bioaccessibility (%)
	Non-Digested	Digested	
**Anthocyanins**			
Kuromanin	72.9 ± 0.1	15.1 ± 0.04	20.71
Pelargonidin-3-*O*-glucoside	21.8 ± 0.2	1.1 ± 0.01	5.05
**Phenolic acids**			
Gallic acid	12.7 ± 0.1	35.3 ± 0.02	177.0
Protocatechuic acid	29.1 ± 0.1	14.6 ± 0.1	50.17
Coumaric acid	13.6 ± 0.1	6.0 ± 0.04	44.12
Synapic acid	9.1 ± 0.1	2.9 ± 0.01	31.89
Chlorogenic acid	43.9 ± 0.2	16.9 ± 0.1	38.50
Caffeic acid	n.d.	2.3 ± 0.2	/
Ferulic acid	11.0 ± 0.1	4.7 ± 0.04	42.73
Syringic acid	11.1 ± 0.1	2.9 ± 0.02	26.13
Ellagic acid	16.3 ± 0.1	11.0 ± 0.1	67.48
**Flavonoids**			
Catechin	12.8 ± 0.2	8.6 ± 0.1	67.19
Myricetin	16.5 ± 0.3	3.8 ± 0.03	23.03
**Total **	**270.8 ± 1.7**	**127.5 ± 1.01**	**47.08**

n.d.–not detected.

**Table 3:** Antimicrobial activity of the 80% ethanolic _(aq)_ raspberry pomace extract (1 mg/mL) against selected foodborne bacteria. Negative control (80% ethanol) is already calculated in results. As a positive control, the tetracycline disk was used. Results are expressed as mean ± SD of duplicate determinations.

	Sample	Inhibition Zone (mm)
Gram-positive bacteria	*Bacillus cereus*	7.78 ± 0.25
	*Staphylococcus aureus*	9.25 ± 0.29
	*Listeria monocytogenes*	7.59 ± 0.69
Gram-negative bacteria	*Escherichia coli*	8.66 ± 0.01
	*Yersinia enterocolitica*	7.13 ± 0.27
	*Klebsiella pneumoniae*	6.91 ± 0.01
	*Pseudomonas aeruginosa*	7.50 ± 0.44
	*Salmonella* Enteritidis	n.e.

n.e.–no effect.

**Table 4:** TPC, TFC, TAC, TTC, and DPPH of digested and non-digested Meringue cookies with raspberry pomace extract and commercial raspberry colorant (control cookie was subtracted). Values are expressed as mean ± standard deviation of three experiments. Data are reported on a dry weight (dw) basis. Different letters in the same line and compound represent statistically different results (*p* ≤ 0.05).

		Meringue Cookie with Raspberry Pomace Extract	Meringue Cookie with Commercial Colorant
Non-digested	TPC (mg GAE/g dw)	8.49 ± 0.74 ^c^	0.74 ± 0.13 ^de^
	TFC (mg CE/g dw)	1.22 ± 0.06 ^a^	0.21 ± 0.02 ^d^
	TTC (mg CE/g dw)	0.71 ± 0.21 ^a^	n.d.
	TAC (mg CGE/100 g dw)	2.67 ± 0.12 ^c^	0.09 ± 0.01 ^f^
	DPPH (% RSA)	30.31 ± 6.6 ^c^	7.46 ± 1.6 ^ef^
Oral phase	TPC (mg GAE/g dw)	7.58 ± 0.53 ^c^	1.38 ± 0.11 ^de^
	TFC (mg CE/g dw)	0.53 ± 0.07 ^c^	0.06 ± 0.01 ^f^
	TTC (mg CE/g dw)	0.51 ± 0.02 ^b^	n.d.
	TAC (mg CGE/100 g dw)	2.77 ± 0.61 ^c^	n.d.
	DPPH (% RSA)	29.83 ± 1.60 ^c^	6.31 ± 0.32 ^f^
Gastric phase	TPC (mg GAE/g dw)	9.09 ± 1.22 ^b^	0.95 ± 0.40 ^d^
	TFC (mg CE/g dw)	0.52 ± 0.05 ^c^	0.04 ± 0.01 ^f^
	TTC (mg CE/g dw)	0.27 ± 0.006 ^c^	n.d.
	TAC (mg CGE/100 g dw)	3.62 ± 0.22 ^b^	0.46 ± 0.02 ^d^
	DPPH (% RSA)	47.99 ± 2.9 ^a^	12.62 ± 0.20 ^d^
Intestinal phase	TPC (mg GAE/g dw)	10.74 ± 1.60 ^a^	2.9 ± 0.43 ^b^
	TFC (mg CE/g dw)	0.72 ± 0.02 ^b^	0.17 ± 0.01 ^e^
	TTC (mg CE/g dw)	0.21 ± 0.007 ^c^	n.d.
	TAC (mg CGE/100 g dw)	8.27 ± 0.41 ^a^	1.05 ± 0.21 ^d^
	DPPH (% RSA)	37.66 ± 3.50 ^b^	9.46 ± 0.90 ^e^

n.d.–not detected. TPC: total phenolic content; TFC: total flavonoid content; TAC: total monomeric anthocyanin content; TTC: total condensed tannin content GAE: gallic acid equivalent; CE: catechin equivalent; CGE: cyanidin-3-*O*-glucoside equivalent.

## Data Availability

The data supporting the findings of this study are available within the article.

## Supplementary Materials

The following supporting information can be downloaded at https://www.mdpi.com/article/10.3390/plants14152444/s1, Figure S1: Chromatogram of a non-digested raspberry pomace extract (black trace) and of the same extract after intestinal phase of digestion (blue trace). Compounds: 1-Gallic acid; 2-Syringic acid; 3-Chlorogenic acid; 4-Myrcetin; 5-Caffeic acid; 6-Kuromanin; 7- Coumaric acid; 8-Ellagic acid; 9-Ferulic acid; 10-Pelargonidin-3-*O*-glucoside; 11-Protocatechuic acid; 12-Snapic acid; 13-Catechin; Table S1: The antioxidant activity (FRAP assay) expressed as μmol Fe^2+^/g dw of raspberry pomace, meringue cookie (MC) with raspberry pomace and MC with commercial aroma; Table S2: Thermal degradation of phenolic compounds from the extract and from the fortified meringue cookie. Method S1: Antioxidant activity—FRAP assay. Ref. [50] is cited in the Supplementary Materials.
