# Supplementary material for: Hydroethanolic Extracts of Raspberry (Rubus idaeus) Pomace as Ingredients of Functional Foods: Characterization and Effect of Gastrointestinal Digestion
Source: Plants (Basel). 2025 Aug 7;14(15):2444. doi: 10.3390/plants14152444 (PMC12349386; doi:10.3390/plants14152444)
Supplement: Supplementary file 1 [file plants-14-02444-s001.zip › plants-3773748-supplementary.pdf]

SUPPLEMENTARY MATERIAL

Hydroethanolic extracts of raspberry (*Rubus idaeus*) pomace as ingredients of functional foods: characterization and effect of gastrointestinal digestion

Ziva Vipotnik [1], Majda Golob [2] and Alen Albreht [1,*]

[1]Laboratory for Food Chemistry, Department of Analytical Chemistry, National Institute of Chemistry, Hajdrihova 19, Ljubljana, SI-1000, Slovenia; ziva.vipotnik@ki.si; alen.albreht@ki.si

[2]Institute of Microbiology and Parasitology, Veterinary Faculty, University of Ljubljana, Gerbičeva ulica 60, Ljubljana, SI-1000, Slovenia; majda.golob@vf.uni-lj.si

* Correspondence: alen.albreht@ki.si; Tel.: +386 14760269

SUPPLEMENTARY METHODS

Method S1. Antioxidant activity – FRAP assay

Ferric reducing antioxidant power (FRAP assay) was performed as described Meneses et al. [1]. The absorbance is determined at 593 nm and an aqueous solution of ferrous sulfate was used to build the calibration curve. FRAP values are expressed as micromoles of ferrous equivalent per g of dry weight material (μmol Fe^{2+} /g dw).

SUPPLEMENTARY FIGURES

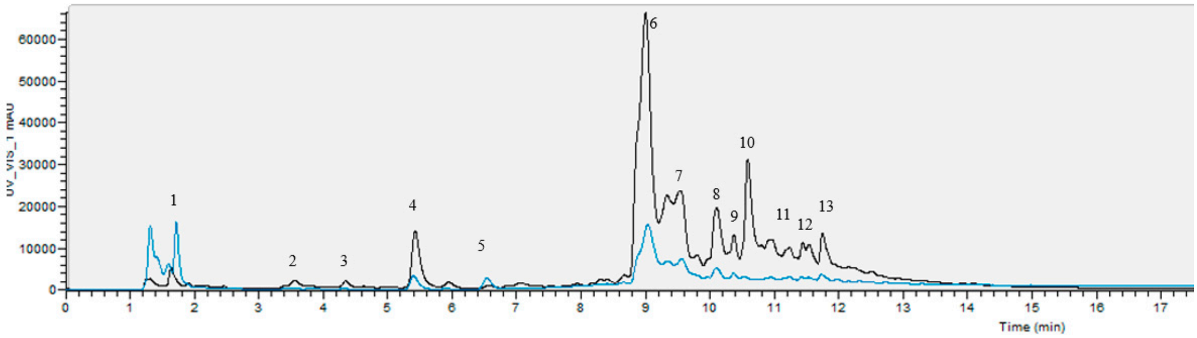

Figure S1. Chromatogram of a non-digested raspberry pomace extract (black trace) and of the same extract after intestinal phase of digestion (blue trace). Compounds: 1-Gallic acid; 2-Syringic acid; 3-Chlorogenic acid; 4-Myrcetin; 5-Caffeic acid; 6-Kuromanin; 7- Coumaric acid; 8-Ellagic acid; 9-Ferulic acid; 10-Pelargonidin-3-*O*-glucoside; 11-Protocatechuic acid; 12-Snapic acid; 13-Catechin

SUPPLEMENTARY TABLES

Table S1. The antioxidant activity (FRAP assay) expressed as μmol Fe^{2+} /g dw of raspberry pomace, meringue cookie (MC) with raspberry pomace and MC with commercial aroma.

	Raspberry pomace	MC – raspberry pomace extract	MC – commercial aroma
**Non-digested**	53.1 ± 0.3	18.9 ± 0.2	5.4 ± 0.01
**Oral**	30.09 ± 0.2	16.5 ± 0.3	5.1 ± 0.04
**Gastric**	11.7 ± 0.3	25.8 ± 0.3	4.8 ± 0.1
**Digested**	8.1 ± 0.4	20.4 ± 0.7	5.1 ± 0.1

Table S2. Thermal degradation of phenolic compounds from the extract and from the fortified meringue cookie.

	Raspberry pomace extract			Meringue cookie + extract		
	**Untreated**	**70 °C; 2 h**	**% recovery**	**Untreated**	**70 °C; 2 h**	**% recovery**
**TPC** (mg GAE/g dw)	472.9 ± 0.1	345 ± 5	73	12 ± 1	8.5 ± 0.7	71
**TFC** (mg CE/g dw)	15.5 ± 0.1	13.1 ± 0.6	85	1.2 ± 0.4	1.22 ± 0.06	102
**TTC** (mg CE/g dw)	2.6 ± 0.2	2.4 ± 0.3	92	1.3 ± 0.7	0.7 ± 0.2	54
**TAC** (mg CGE/100g dw)	73 ± 1	*n.d.*	0	2.9 ± 0.4	2.7 ± 0.1	93

n.d. – not detected

REFERENCES:

[50] Meneses, N. G. T.; Martins, S.; Teixeira, J. A.; Mussatto, S. I. Influence of extraction solvents on the recovery of antioxidant phenolic compounds from brewer's spent grains. *Sep. Purif. Technol.* **2013**, *108*, 152–158, https://doi.org/10.1016/j.seppur.2013.02.015.
